# Crystal structure of the signaling helix coiled-coil domain of the β1 subunit of the soluble guanylyl cyclase

**DOI:** 10.1186/1472-6807-10-2

**Published:** 2010-01-27

**Authors:** Xiaolei Ma, Annie Beuve, Focco van den Akker

**Affiliations:** 1 Department of Biochemistry/RT500, Case Western Reserve University, 10900 Euclid Ave. Cleveland, OH 44106, USA; 2 Department of Pharmacology and Physiology, UMDNJ - New Jersey Medical School Medical Sciences Building, I655/I664 185 S. Orange Avenue, Newark, NJ 07103, USA

## Abstract

**Background:**

The soluble guanylyl cyclase (sGC) is a heterodimeric enzyme that, upon activation by nitric oxide, stimulates the production of the second messenger cGMP. Each sGC subunit harbor four domains three of which are used for heterodimerization: H-NOXA/H-NOBA domain, coiled-coil domain (CC), and catalytic guanylyl cyclase domain. The CC domain has previously been postulated to be part of a larger CC family termed the signaling helix (S-helix) family. Homodimers of sGC have also been observed but are not functionally active yet are likely transient awaiting their intended heterodimeric partner.

**Results:**

To investigate the structure of the CC S-helix region, we crystallized and determined the structure of the CC domain of the sGCβ1 subunit comprising residues 348-409. The crystal structure was refined to 2.15 Å resolution.

**Conclusions:**

The CC structure of sGCβ1 revealed a tetrameric arrangement comprised of a dimer of CC dimers. Each monomer is comprised of a long a-helix, a turn near residue P399, and a short second a-helix. The CC structure also offers insights as to how sGC homodimers are not as stable as (functionally) active heterodimers via a possible role for inter-helix salt-bridge formation. The structure also yielded insights into the residues involved in dimerization. In addition, the CC region is also known to harbor a number of congenital and man-made mutations in both membrane and soluble guanylyl cyclases and those function-affecting mutations have been mapped onto the CC structure. This mutant analysis indicated an importance for not only certain dimerization residue positions, but also an important role for other faces of the CC dimer which might perhaps interact with adjacent domains. Our results also extend beyond guanylyl cyclases as the CC structure is, to our knowledge, the first S-helix structure and serves as a model for all S-helix containing family members.

## Background

**Figure 1:**
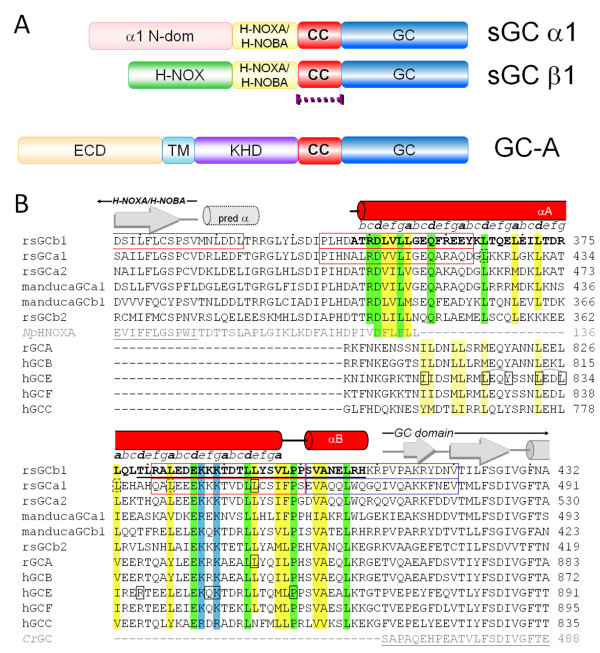
**Guanylyl cyclase domain structure and structure-based sequence alignment**. *A*, domain organization of CC containing sGC and mGCs (crystallized part in purple dotted line). *B*, Structure-based sequence alignment of CC and adjacent regions in rat sGCβ1, sGCα1, sGCα2, Manduca sexta sGCα1 and sGCβ1, rat sGCβ2, rat GC-A, and human GC-B, GC-E, GC-F, and GC-C. Also included are Npun02000820 of *Nostoc punctiforme *PCC73102, labeled *Np*HNOXA, and the guanylyl cyclase of *Chlamydomonas reinhardtii*, labeled *Cr*GC (in grey) to indicate the proximity of the CC flanking H-NOXA and GC domains, respectively, as their structures have been determined [5,10] (residues included in the structures are grey underlined with grey secondary structure elements). The sequence of the crystallized sGCβ1 CC is bold with red secondary structure elements. Completely conserved residues in the sGCs are highlighted green with possible conservation extending into mGCs. Mostly conserved hydrophobic residues are in yellow; mostly conserved basic residues are in blue. sGCβ1 residues 379-408 found to be important for dimerization [22] are black underlined. sGCβ1 residues 344-363, 381-400, and sGCα1 440-459 are important for sGC dimerization (red box); sGCβ1 401-420 and sGCα1 460-479 are important for activity but not dimerization (blue box) [24]. The *a-d *designation based on the CC crystal structure A:B dimer is generated using SOCKET [27] and slightly extended at the (frayed) ends. CC mutations [23,28,40-43] are shown in a black box. Secondary structure predictions http://npsa-pbil.ibcp.fr/cgi-bin/npsa_automat.pl?page=/NPSA/npsa_seccons.html of all 7 top sequences suggested a small helix present between the H-NOXA/PAS and αA helix (black dotted lines).

Mammalian guanylyl cyclases are key signaling proteins that produce the second messenger cGMP thereby regulating a variety of different processes such as vasodilation, diuresis, vision, and bone growth [1]. These cyclases are either membrane bound or are found as soluble forms. Members of the membrane guanylyl cyclases (mGC) include the atrial natriuretic peptide receptor (GC-A), heat-stable enterotoxin receptor (GC-C), and retinal guanylyl cyclases (GC-E and -F) whereas the soluble version is known as the soluble guanylyl cyclase (sGC). cGMP produced by these guanylyl cyclases activates downstream signaling proteins such as cGMP-dependent kinases and cGMP-dependent ion channels [2]. The cyclases are all activated by different ligands that are in most cases recognized by the N-terminal portion of the cyclases. Therefore, this N-terminal region is quite divergent amongst the different cyclases [1]. In contrast, the C-terminal region of all the cyclases have two domains found in all mammalian guanylyl cyclases: the coiled-coil domain (CC) and the adjacent C-terminal catalytic guanylyl cyclase domain (GC)(Figure 1). Considerable progress has been made on the structural characterization of domains of the receptors, or homologs thereof. These domains include the GC-A receptor hormone-binding domain [3], homologous catalytic guanylyl cyclase domains [4,5], and domains that are homologous to sGC: ligand binding heme-nitric-oxide-and-oxygen binding domains (H-NOX or also termed H-NOB) [6-9], and the H-NOXA/H-NOBA/PAS domain [6,10]. One of the guanylyl cyclase domains that has yet to be structurally characterized is the CC domain and that is the focus of this study.

The mammalian sGC is a heterodimer with an α and β subunits. Each subunit has two isoforms, α1 α2 β1 β2, yet the sGCα1β1 is the most abundant whereas the sGCα2β1 is more predominant in brain tissue [11]. The precise role for β2 subunit is not fully understood and it could have a dominant negative regulatory role [12]. The subunit arrangement for sGCβ1 includes the above mentioned C-terminal CC and GC domains as well as an N-terminal NO-sensing H-NOX/H-NOB and adjacent H-NOXA/H-NOBA/PAS domains. sGCα1 is 30% sequence identical to sGCβ1 and has a similar subunit organization except that its N-terminal domain does not contain a heme (Figure 1).

Besides heterodimerization, homodimers of sGC homodimers have also been observed for β1[13], β2 [14], and the less stable α1 homodimer [15]. sGC homodimers are not active [13], except for the *Manduca sexta*'s *β*3 subunit [16] and the sGC β2 subunit although the latter's needs non-physiological manganese indicating that it might not be active and/or dimerized under physiological conditions [14]. The homo- and heterodimeric forms of sGC are thought to be in a physiological equilibrium [13] with heterodimerization being preferred whereas homodimeric β1β1 [17] and α1α1 are found to be unstable *in vivo *[18]. Understanding the underlying reason for the instability of homodimeric sGC is important as its subunit expression levels are known to change [19-21] which could lead to uneven subunit levels resulting in unstable sGC homodimers thereby further affecting subunit levels. Dimerization within sGC is mediated by at least three inter-domain interactions involving the GC, H-NOXA/H-NOBA, and CC subdomains. The latter two dimerization interactions were found to be the most pronounced and include β1 residues 204-244 (in H-NOXA domain) and residues 379-408 (in CC domain)[22]; the corresponding regions in sGCα1 are also critical for sGC activity [23]. Recent studies have narrowed down these dimerization regions via deletion studies [24](Figure 1B) and even structurally characterized an H-NOXA dimer [10] leaving the CC region as the only region to be structurally explored.

The sequence of the CC region of guanylyl cyclases are relatively conserved and are postulated to be part of a larger class called the signaling helix (S-helix) [25]. This recent *in silico *study identifies this novel signaling module in between diverse N-terminal sensory domains and various C-terminal catalytic domains ranging from histidine kinases, PP2C phosphatases, NtrC-like AAA+ ATPases, diguanylate cyclases to guanylyl cyclases. Although members in this S-helix family share limited but detectable sequence identity among each other, their secondary structures are predicted to be entirely helical. To our knowledge, no S-helix structure has yet been determined so the elucidation of sGC CC S-helix domain would not only contribute to the understanding of guanylyl cyclase hetero- vs homodimerization, activation, domains cross-talk but also provides a structural prototype for the entire S-helix family. We present here the 2.15 Å crystal structure of the CC region of the sGCβ1.

## Results

### sGCβ1 CC construct form oligomers in solution

**Figure 2:**
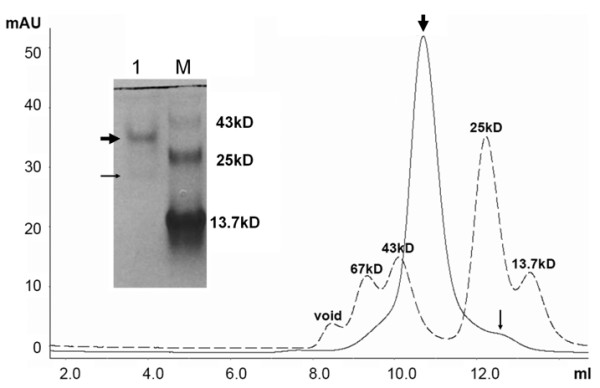
**Oligomerization state analysis of sGC CC domain**. Size exclusion gel filtration chromatography of the CC sGCβ1 using a Superdex 75 column resulted in an oligomeric 38.5 kD species (thick arrow) and a minor 19.5 kD species (thin arrow). The column was calibrated using a mixture of different Mw standards (interrupted line). *Inset*, Native polyacrylamide gel electrophoresis analysis of CC sGCβ1 (lane 1) and a mixture of 3 Mw standards (lane M) revealing a major and minor species with similar Mw as obtained by size-exclusion chromatography (thick and thin arrow, respectively).

The N- and C-terminal boundaries of the *Rattus norvegicus *sGC 348-409 CC domain were chosen such that it starts after the PAS-like/H-NOXA domain and ends before the cyclase domain (based on sequence and secondary structure analysis). This sGC CC monomer has a theoretical pI of 5.62 and a calculated molecular weight of 7712.86Da. We performed analytic gel filtration of the CC construct prior to crystallization experiments to estimate the oligomerization state of this construct (Figure 2). The majority of the CC protein eluted as a 38.5 kD protein and minor portion as a 19.5 kD protein. Taking into account the non-globular nature of a coiled-coil protein, which makes the protein appear with a larger molecular weight, we assume that the majority of the protein is tetrameric with a minor dimeric species present. Similar results were obtained with native polyacrylamide gel electrophoresis analysis (Figure 2). Efforts regarding the corresponding CC domain in sGCα1 to arrive at a heterodimeric CC complex were not successful due to inability of the CC of sGCα1 to be expressed.

### The sGCβ1 CC monomers form an anti-parallel four-helix bundle in crystal

**Figure 3:**
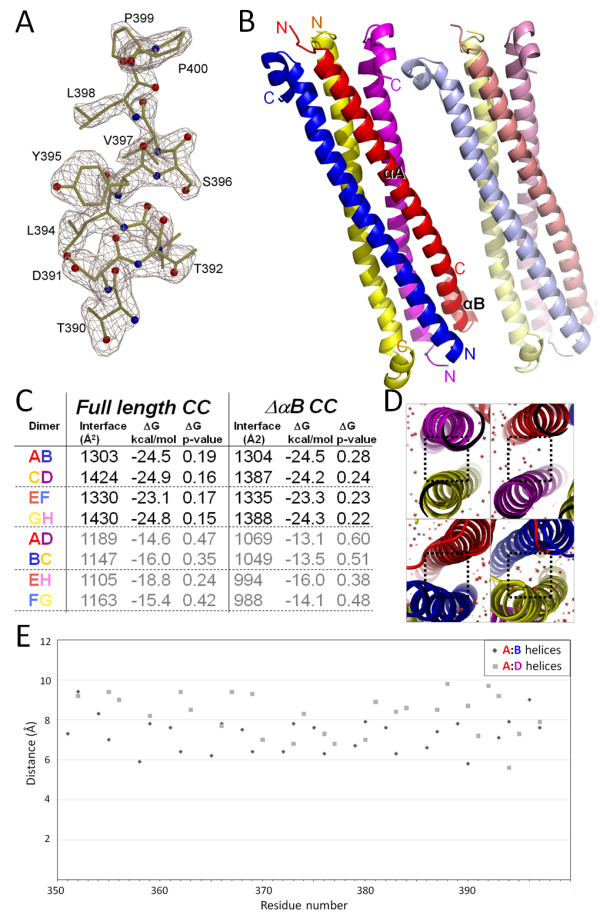
**Structure of CC of sGCβ1 and dimer analysis**. *A*, Omit | F_o_| -| F_c_| electron density of the C-terminal end of the αB helix of molecule B, contoured at 2.5 σ. *B*, schematic diagram of asymmetric unit contents of CC of sGCβ1 revealing 2 CC tetramers. Shown are CC molecule A (red) with the long αA and short αB helix labeled, as well as molecules B (blue), C (yellow), D magenta, E (light red), F (light blue), G (light yellow), and H (light magenta). *C*, dimer interface calculations using PISA [26], *D*, different dimer interfaces and the presence or absence of water molecules (red crosses) between monomers. *E*, graph depicting Cα-Cα distance for αA residues from molecule A to either the nearest residue in molecule B (diamonds) or to molecule D (grey squares) that are shorter than 10 Å

**Figure 4:**
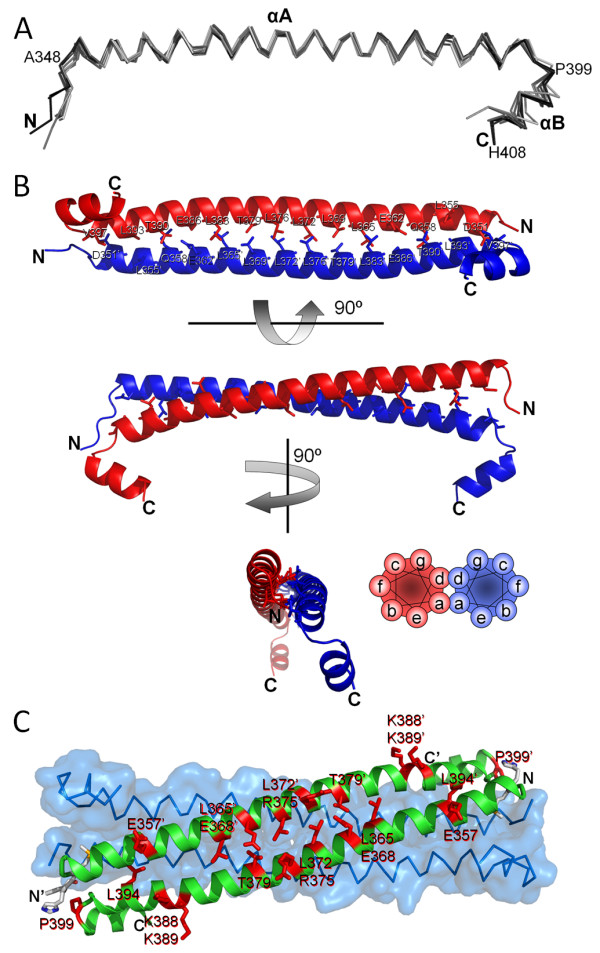
**Structures of the monomer and dimer organization of CC sGCβ1**. *A*, superposition of the 8 CC monomers. *B*, three different views of the AB dimer. Depicted also are the a-d residues at the dimer interface. Schematic diagram of anti-parallel CC dimer including a-g residue assignment is included for reference to illustrate a-d appearance at the interface. *C*, sites of function-affecting mutations present in the CC region of sGCs or mGCs are mapped onto the CC dimer sGCβ1 structure. The second dimer, as part of the tetramer, is also depicted in a Cα trace with a transparent solid surface.

We determined the crystal structure of the *Rattus norvegicus *sGC CC domain (residues A348-K409) to 2.15 Å resolution using SeMet single-anomalous-dispersion (SAD)-phasing (Table 1). Crystals were initially obtained of the *wt *sequence but the SAD phasing required the introduction of an additional Met residue via the I371 M mutation. There are 8 CC molecules in the asymmetric unit and each monomer contains a long amphipathic a helix aA with 13 helical turns followed by a turn and a short helix aB (Figure 3). The turn is initiated by a conserved proline residue, P399 (Figure 1B, 3A, and 4). Four sGC CC monomers come close together to form an apparent four-helix bundle in an anti-parallel arrangement with two of such tetramers in the asymmetric unit (Figure 3). The overall dimensions of each tetramer is about 85 × 37 × 25 Å^3^. The rsGC CC domain is quite charged as 1/3 of its residues are either acidic or basic (12 D/E and 9 K/R). The eight monomers are conformationally similar to each other, with root-mean-square deviation (r.m.s.d.) for Ca atoms ranging between 0.27 Å and 1.50 Å based on pair-wise structural comparisons (Figure 4A). Some of the (four) N-terminal residues, introduced as cloning artifacts (GSHM-) were ordered for each monomer. The two tetramers in the asymmetric unit are similar as a superposition of the two CC 'tetramers' results in a RMSD of 1.04 Å for 252 Cα atoms. The I371 M mutation that was generated for SeMet phasing is not involved in the dimer or tetramer interface. Note that the site of the I371 M mutation was chosen carefully as to not to interfere with the predicted a-d pattern of CC dimerization. The I at position 371 is also not fully conserved in sGCβ1 and can also be a V (such as *Manduca sexta*, see Figure 1), or M (in drosophila) suggesting that its mutation to M can be accommodated and will likely not cause a negative effect on dimerization and/or coiled-coil interactions.

**Table 1:** X-ray data collection, phasing and refinement statistics

**Data collection**	**Se-Peak**
Wavelength (Å)	0.97926
Space group	C2
Cell dimension	a = 152.039 Å
	b = 65.814 Å
	c = 98.626 Å
	β = 129.948°
Resolution (Å)	50.0-2.15 (2.23-2.15)
Total observations	298063
Unique observations	78615
I/SigI	10.9 (2.1)
Redundancy	3.8 (3.8)
Completeness	99.2 (99.5)
R_sym_^a ^(%)	8.2 (34.6)
**SAD phasing**	
Resolution	50.0-2.2
No. of Se sites^b^	14
FOM_SOLVE_^c^	0.3
FOM_RESOLVE_^d^	0.64
**Refinement**	
Resolution (Å)	50.0-2.15
No. of protein atoms	4152
No. of waters	460
R_work _(%)	21.3
R_free_(%)	26.1
RMSD bond length (Å)	0.010
RMSD bond angles (°)	1.21
**Ramachandran plot**	
Most favoured (%)	98.3
Additionally allowed (%)	1.5
Generously allowed (%)	0.2
Disallowed (%)	0.0

Values in parentheses represent the highest resolution shell

^a^*R*_sym_(*I*) = Σ_*hkl*_Σ_*i*_|*I*_*i*_(*hkl*) - ⟨*I*(*hkl*)⟩|/Σ_*hkl*_Σ_*i*_*I*_*i*_(*hkl*) where the summations are over *i *observations of each reflections and all *hkl*. ⟨*I*(*hkl*)⟩ is the average intensity of the *i *observations. *R*_work _= |*F*_(obs)_-*F*_(calc)_|/*F*_(obs)_

^b^Number of selenium sites located by SOLVE

^c^Figure of merit calculated from SOLVE

^d^Figure of merit calculated from RESOLVE

^e^R_free _is calculated for 5% of randomly selected reflections not used in the refinement

### Inter-monomer analysis of sGCβ1 tetramer: dimer of dimers

The interactions between the CC monomers within a tetramer were analyzed using the PISA protein-interface webserver [26]. The analysis revealed that the interface buried between certain sets of monomer-monomers is larger compared to others. The AB, CD, EF, and GH interfaces are all larger compared to the AD, BC, EH, and FH interfaces (Figure 3C). These differences were even more pronounced for PISA's calculated ΔG and ΔG-P-value (Figure 3C) regarding these dimer interfaces (P-values significantly below 0.5 indicate that a relative higher percentage of the buried surface is hydrophobic indicative of a biologically-relevant interface). Furthermore, if the short aB helix is removed from the calculations, since it is not part of the long coiled-coil helix, the differences are even larger (ΔαB CC values in Figure 3C). The AB and CD interface are packed tighter as well and therefore also have no waters present whereas the AD and BC interfaces do have water molecules present (Figure 3D). The tighter packing is evidenced as the Ca-Ca distances between Ca's in A and nearest Ca in the B helix compared to the A and D helices are generally larger for the A-D pair (Figure 3E): the average Ca-Ca distances for A:B helices (ignoring distances > 10 Å) is 7.3 Å and for the A:D helices is 8.3 Å.

These different analyses indicate that the tetrameric CC organization observed is likely comprised of a dimer (Figure 4) of dimers and that the non-physiological tetramer formation can perhaps be attributed to either the absence of the sGCa1 CC or perhaps the presence of the N-terminal GSHM cloning residues of which the non-native methionine (M347) is located at the tetramer interface (Figure 4C). This dimer of dimers interpretation is in agreement with the CC dimer being also observed as a minor dimeric species in solution (Figure 2). Since the expected CC oligomerization state of guanylyl cyclases is dimeric, we narrow down our subsequent analysis to mainly the CC dimer.

### Dimer analysis of sGCβ1 CC

The CC monomers A and B (and the equivalent CD, EF, and GH) form an anti-parallel CC dimer (Figure 4B). The side-chain directions of a-helices follow a heptad (a-g) repeat in that residue *i *and *i*+7 face the same direction. The CC dimer interface is formed predominantly via the a-d pattern residues and the a-d assignment, as obtained from SOCKET [27], is indicated in Figure 1B. A large number of the a-d residues are hydrophobic which is the characteristic feature for CC domains. Our observed a-d pattern was also predicted by homology modeling of a S-helix [25] whereas the CC of GC-E was modeled to have a different a-d pattern [28]. Note that the observed a-d pattern for sGCβ1 can also be predicted from the sequence alone using PCOILS [29] which predicted the same a-d assignment for residues Y363-K389 with probability of higher than 0.75. However, the Marcoil prediction server [30] yielded a different a-d assignments. These possible ambivalences in a-d assignments could be used by the CC domain for signaling perhaps via rotation of helices as is also postulated to occur in the HAMP domain [31].

The CC of sGC harbors known regions found to be critical for dimerization. These include the broadly defined sGCa1 367-462 [15] and sGCβ1 379-408 [22] stretches (Figure 1B). A recent deletion study narrowed these regions down to sGCβ1 344-363 and 381-400 as well as sGCa1 440-459 which were all found to be important for dimerization [24]. sGCβ1 401-420 and sGCa1 460-479 were found to be important for activity but not for dimerization [24](Figure 1B). The results from this deletion study are in agreement with our CC dimer structure since the latter two regions (which includes aB helix) are not found at the dimer interface whereas the other ones are at the dimer interface since they are part of the long aA helix.

The CC domain is found in both mGCs and sGC and other S-helix containing proteins [25]. The sequence alignment shows considerable sequence conservation, in particular in the C-terminal half of the S-helix (Figure 1B). The helix distance plot (Figure 3E) shows the distance between the aA helices in the middle of the CC A:B dimer is closer compared to the distances near the ends of the helices.

### CC orientation

The observation that the CC dimeric arrangement is anti-parallel is somewhat unexpected since other groups had suggested a parallel arrangement for guanylyl cyclase CC domains after having carried out molecular modeling studies of a parallel CC dimer [25,28]. Our homo-oligomer CC sGCβ1 structure is representative of a physiologically present homodimeric sGCβ1β1 which, in the full length sGC, was found to be not stable. Our structure does not represent the active sGCa1β1 since it was missing its heterodimeric sGCa1 CC partner in the crystallization experiment. We therefore explored whether a parallel arrangement was also a possibility in the presence of sGCa1 CC. CC's either parallel or anti-parallel orientation possibilities were investigated by considering the possibilities of salt-bridge formation, residue preference at a-d positions, and inter-domain constraints.

#### -Potential salt-bridge formation

Whereas the a-d positions of a CC are favored to have leucine-like residues to promote CC formation, the residues at the e and g positions can be found to make inter-helix salt-bridges [32,33]: parallel CCs are observed to form g-e' salt-bridges whereas anti-parallel CCs can form g-g' and e-e' salt-bridges. To analyze the possible formations of such salt-bridges in both orientations for both homodimeric sGCβ1 and heterodimer sGCa1β1, we generated models of the different possible CCs. To generate an anti-parallel heterodimeric sGCa1β1 aA CC dimer, we used COOT [34] to mutate the residues of one of the monomers to the sGCa1 sequence using the alignment in Figure 1B. To obtain a parallel sGCβ1 homodimer, we used the coordinates of the parallel RhoKinase CC dimer [35](PDBid 1UIX) as template for superimposing the sGCβ1 CC using the a-d residue assignment as guide. This generated homodimeric CC model was used to make a heterodimeric parallel CC model via changing the residues of one of the monomers into the sGCa1 sequence using COOT. These 3 models of aA helix CC dimers were not refined using minimization methods as they merely serve the purpose of visualizing the possibilities of salt-bridge formation of residues at the e and g positions. The following salt-bridges are possible for CC homo-/heterodimers in either a parallel or anti-parallel CC orientation:

-Anti-parallel heterodimer g-g' or e-e' salt-bridges: 1 (sGCa1 K448-sGCβ1 E361)

-Anti-parallel homodimer g-g' or e-e' salt-bridges: 4 (sGCβ1 E368-sGCβ1 R375; sGCβ1 E361-sGCβ1 K389 and their two symmetry related ones)

-Parallel heterodimer g-e' salt-bridges: 2 (sGCa1 K432: sGCβ1 E368, sGCa1 K425-sGCβ1 E361).

-Parallel homodimer g-e' salt-bridges: 0

This analysis suggests that only in the parallel CC orientation is there a possible electrostatic attraction benefit of a1β1 heterodimer formation compared to β1β1 homodimer formation (number of salt-bridges can increase from 0 to 2).

#### -L, V, I, N residue preferences

In addition to electrostatic attraction, the presence of leucine, valine, isoleucine, and asparagine residues at either a or d positions can yield clues regarding the parallel or anti-parallel nature of CCs. Based on the analyses of previous dimeric CC structures, the L_d_:L_a _ratio for parallel was found to be 3.5 but was 1.2 for anti-parallel CCs [36]. In addition, the same study found that for the branched residues valine and isoleucine, the ratio of (V+I)_a_:(V+I)_d _was 14.2 for parallel and only 1.8 for anti-parallel. This ratio is also in agreement with that the isoleucine residue was found to be difficult to be accommodated at the d position for parallel CCs [37]. Finally, parallel CCs were found to have a 4-fold preference for asparagine residue at the a position compared to anti-parallel CCs [27]. To investigate whether these a/d position preferences can aid in suggesting whether CCs from sGCs and mGCs are parallel or anti-parallel, we analyzed their a-d appearance using the sequence alignment in Figure 1B as well as an expanded sGC alignment including 15 sGC-only sequences (results listed in brackets): L_d_:L_a _= 32:23 (45:42), (V+I)_a_:(V+I)_d _= 17:0 (22:0), and N_a_:N_d _= 1:0 (3:0). These results indicate that the a:d appearance of three of the four analyzed residues (V, I, and N) suggest that the CC orientation could be parallel (the L is less conclusive).

#### -Inter-domain distance constraints

**Figure 5:**
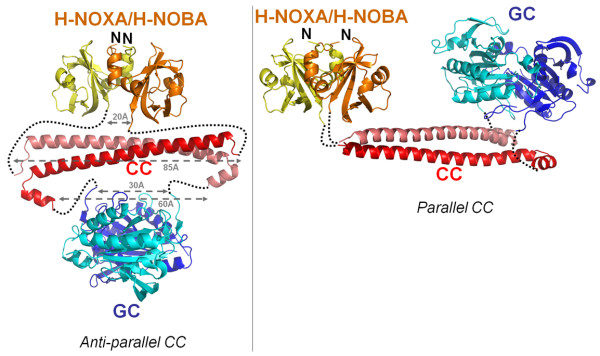
**organization of heterodimeric sGC based on CC orientation**. Possible H-NOXA/CC/GC organization based on an anti-parallel CC dimer (left) and based on a parallel CC dimer (right). The H-NOXA/H-NOBA (yellow), CC (red), and GC (blue) domains are depicted. Distances of C-terminus:C-terminus and N-terminus:N-terminus within either dimeric H-NOXA/H-NOBA, CC, or GC are shown using grey arrows.

Finally, an additional clue regarding parallel *vs *anti-parallel CCs can come from the fact that these different orientations would have different distances between their termini which have to still somehow connect to their adjacent domains via intervening residues. We therefore constructed partial composite models of the H-NOXA/CC/GC domains with in one case a parallel and the other an anti-parallel CC orientation (Figure 5). The H-NOXA dimer and the GC dimer are taken from recently determined crystal structures [5,10] and part of their sequence is also included in the sequence alignment to show their sequence proximity to the CC (Figure 1B). The sequence identity between the *Ns *HNOXA and the sGCβ1 H-NOXA domain is 35% and the identity between the GC domains of sGCβ1 and the mammalian *Chlamydomonas reinhardtii *GC structure is 42%. In the anti-parallel arrangement, the distance between the two N-termini of the CC dimer is ~85 Å whereas the distance between the two C-termini of the H-NOXA dimer is only ~20 Å (Figure 5). These differences indicate that each connection would need to stretch about (85-20)/2 = 32.5 Å. This distance is rather large but can perhaps be reached via the 20 intervening residues between these domains (Figure 1B) as each Ca-Ca distance of adjacent residues is ~4 Å. Regarding the other inter-domain connections, the distance between the anti-parallel CC C-termini is 60 Å whereas the distance between the N-termini of the GC dimer is only 30 Å (Figure 5). This indicates that each linker would need to stretch about (60-30)/2 = 15 Å which is too large for having only 2 intervening residues between CC and GC (Figure 1B). These calculations are of course dependent on that either the H-NOXA or GC dimer does not change globally (or locally) during activation but both of those have either been speculated to possibly change (for H-NOXA [10]) or observed to change (for a homologous adenylyl cyclase dimer [38]). With the parallel CC arrangement, these inter-domain N- and C-termini distances are shorter and therefore more compatible (Figure 5). It should however be noted that the termini in proteins structures are often flexible thereby possibly changing these distance-based extrapolations. Based on the above arguments indicating that the sGCa1βb1 CC is likely parallel, we were able to expand the partial (CC parallel) composite model of the heterodimeric sGC by adding in the H-NOX coordinates [9] and positioning this domain such its C-termini and the N-termini of H-NOXA are in close proximity and that H-NOX can also interact with the GC domain (not shown); this latter requirement satisfied the direct H-NOX/GC interaction that has previously been observed [39].

Finally, the fact that the C-terminal half of the CC domain is more conserved across the different cyclases (sGCβ1 residue 376-408; Figure 1B), and likely of different length as also noted earlier [6,25], is also indicative of a parallel helix arrangement as the conserved regions will interact with each other which would not occur in an anti-parallel CC arrangement.

### Mapping of guanylyl cyclase CC mutations

The CC domain present in mGCs and sGCs has been the site of a number of genetic mutations and has also been targeted by mutagenesis to probe structure-function. Our crystal structure of CC domain allows us to now map the sites of these mutations on the CC structure to possibly gain additional structure-function knowledge.

First, the CC domain of sGCa1 of the medeka fish *Oryzias latipes *has been targeted for mutagenesis for structure-function studies [23]. The a1 mutations (with the rat a1 sequence number in parenthesis) L434K (L424) and L445K (L435) each caused a slight decrease in dimerization and no decrease of NO stimulation yet increased basal activity by ~3-fold. The a1 L463K (L453) mutation caused loss of basal and NO stimulated activity and slight decrease in dimerization. Combining these mutations in a triple mutant decreased the dimerization to ~30% of wt; adding L452K (L442) mutant to form a quadruple mutant dropped the dimerization to only 10% of wt. Secondly, the retinal guanylyl cyclase GC-E can harbor several blindness causing mutations in the CC domain (R838 [28,28], P858 [40], the double mutation Q847L/K848Q [41], and I816S [42]). In addition, either of the GC-E double mutants L824S/Y827S and L831S/L834S affected receptor function [28]. Thirdly, the GC-A receptor also has one mutation (L845R) that is located within the CC and found to cause loss of activity but not loss of dimerization [43]. Note that this latter mutation is equivalent to the *O. latipes *sGCa1 L463K (L453) mutant (Figure 1B) suggesting that this residue is of considerable importance in both mGCs and sGCs. This residue is equivalent to sGCβ1 L394 which is making a 4 Å van der Waals interaction with L355. The above mentioned function-affecting CC mutations are listed in the sequence alignment (Figure 1B) and their equivalent residues in sGCβ1 are mapped onto their structure (Figure 4). The mutations are roughly evenly distributed along the full length of the CC aA helix as there appears to be no clear concentration of mutations. Furthermore, not all mutations correspond to residues at the a-d positions, indicating a critical functional role for CC regions within and outside the dimerization interface.

## Discussion

We have determined the structure of the CC domain of sGCβ1 which revealed a long aA helix, a short turn, followed by a short aB helix. The oligomeric state at first inspection seems to reveal a tetrameric anti-parallel arrangement yet our detailed analysis suggests that the dimer-of-dimers tetrameric arrangement is non-physiological and likely due to the hydrophobic end regions of the CC normally preferring to interact with other sGC protein regions that are missing in our crystallized construct. These hydrophobic CC end regions ended up therefore packing against another CC dimer to form this non-physiological tetramer. Within the CC dimer structure, we argue that the observed anti-parallel nature of the CC arrangement in the sGCβ1β1 homodimer is likely different from that in full length heterodimeric sGC since its sGCa1 CC partner is missing. In addition to having analyzed the possibilities of salt-bridge formation, residue preference at a-d positions, and inter-domain constraints regarding parallel vs anti-parallel CC formation for the S-helix, additional analysis also suggests a parallel arrangement as will be discussed next.

The S-helix is found in other non-guanylyl cyclase domain containing proteins and its proximity to other domains could also yield insights into the orientation arrangement since the distance of the termini is very different for the two different orientations. For example, the S-helix is also found in front of a diguanylate cyclase domain ([25] and this dimeric protein has an N-terminus-N-terminus distance of ~25 Å (PDBid 1W25[44], which is more compatible with a parallel CC dimer. In addition, the S-helix is often found N-terminal to a DHp domain or N-terminal and C-terminal of the HAMP domain [25]. Both the DHp and HAMP domain are 4-helix bundle dimerized domains with their N-terminus-N-terminus or C-terminus-C-terminus distances being as short as 8, 14, or 38 Å (PDBids 2ASW and 3D36). Anantharaman *et al*. had therefore previously suggested that the S-helix could perhaps merge/extend from the termini of these HAMP and DNp domains [25] which would only be possible in a parallel CC arrangement. In summary, the CC/S-helix region found in cyclases and other proteins is very likely in a parallel arrangement yet the observed anti-parallel CC in the sGCβ1β1 is likely physiological relevant as will be discussed next.

The sGCβ1 homodimer CC structure revealed an anti-parallel arrangement of the monomers and might shed some light into how sGC has evolved to favor heterodimerization over homodimerization (with homodimers not even being very stable). sGC has evolved this characteristic despite that many of the individual subunits are known to homo-dimerize by themselves. The dimerization K_d_'s for the catalytic domain a1a1 and β1β1 homodimers are 10-20 μM and ~6 μM, respectively [39]. Furthermore, sGCβ1 H-NOXA domain homo-dimerization K_d _is less than 60 μM [10] and the β1 CC dimerization is likely also in the μM range. Therefore, having at least three domains each with about a ~10^-5^M homodimerization K_d _constant within one protein would normally yield, due to avidity, an overall homodimerization binding constant in the nM if not pM range. Such an extrapolated strong affinity would normally render the homodimers quite stable and it is therefore surprising that sGC homodimers are found to be unstable [13,17,18]. A possible unifying explanation could perhaps be the non-compatibility of the homo-dimeric CC orientation with respect to the position of the flanking domains: only a parallel CC would align its flanking domains correctly whereas an anti-parallel CC dimer, as observed in our sGCβ1β1 CC structure, might likely not (Figure 5). Such a homo-dimer disfavoring mechanism could be important since only the sGC heterodimer is catalytically functional making it vital for the sGC subunits not to form stable yet unproductive homodimeric complexes. We realize that such a mechanism might be unique to sGC, being heterodimeric, as many other S-helix family members are homodimeric. Perhaps a contributing factor to the uniqueness of part of sGC's homo-dimer disfavoring mechanism could be the possibility of unique 1-residue register slippage changes in the middle of the S-helix [25](Figure 1).

### Conformational changes upon activation

CCs are known to be quite sensitive as small changes to CCs have been known to affect oligomerization and helix orientation (reviewed in [37]). The CC region in guanylyl cyclases is likely also not a static coiled-coil dimer as it has been postulated to be important for regulation/signaling. This possibly regulatory role for the CC was observed in mutagenesis and modeling studies for GC-E indicating that this region was not optimized for dimerization but more for regulation as R838 mutations increased activity and might structurally extend/lengthen the CC region [28]. A bioinformatics study also suggested a regulatory/signaling role for the CC region and has termed this region in cyclases a signaling or S-helix [25]. Both of these studies have indicated the S-helix to be parallel CC and modeled it as such. Some possible CC conformational changes could involve a loop-to-helix transition, as observed in influenza virus hemagglutinin HA2 [45], a shifting/flipping along the interface of the a-d knobs, as modeled for GCN4 [46], or rotation of the helices as evidenced in the HAMP structure [31]. In addition, a helix re-orientation from anti-parallel to parallel or *vice versa *can also not be ruled out.

Despite the above emphasis of the CC in guanylyl cyclases possibly being a signaling module, it is likely that not all signaling of the upstream domains in mGCs and sGCs goes solely through the CC domain to reach the catalytic GC domain. This is evident from that the H-NOX domain can directly interact and inhibit the GC domain in sGC [39].

In addition to possible conformational changes involving just the long aA helix, activation conformational changes could also involve interactions of other sGC regions interacting with the end regions of the CC since there are a number of residues with conserved hydrophobicity (i.e. L352, V353, L354, L394, and L398). These end regions could perhaps interact with either CC's own aB helix, as in our structure since aB folds back on aA, and/or the small helix predicted to be between the H-NOXA and CC domain (see Figure 1B). The CC aA helix could thus use the hydrophobic regions towards its ends that in our sGCβ1 structure form also part of the dimer of dimer interactions. It is noteworthy that the CC ends harbor two conserved residues that are found to be critical for mGC and sGC activation (corresponding to sGCβ1 L394 and P399 as described above in the "Mapping of Guanylyl Cyclase CC Mutations" sections, Figure 1B and 4C) suggesting an important role for this region in receptor activation and/or interactions with the other subdomains of sGC.

Future structural studies are needed to determine the structure of sGC's heterodimeric CC alone and in the intact sGC and what the activation conformational changes are within this region.

## Conclusions

We have determined the crystal structure of the sGCβ1 CC domain to 2.15 Å resolution. This CC structure revealed a long a-helix, a turn near residue P399, followed by a short second a-helix. CC domains are known for their oligomerization behavior and we therefore analyzed the inter-molecule interactions within the asymmetric unit which indicated a dimeric arrangement of the CC sGCβ1 subunits. Additional sequence analysis and modeling of homo- and heterodimeric CCs allowed us to speculate that the hetero-dimerization preference over homo-dimerization of sGC subunits could be, in part, due to inter-helix salt-bridge formation. The CC region has been shown to be a critical region for guanylyl cyclase functioning and is the site for a number of congenital and man-made mutations in both membrane and soluble guanylyl cyclases. The CC sGCβ1 structure allowed mapping of those function-affecting mutations which pointed to an important role for some of the dimerization region but also for residues not involved in the dimer interface. This latter observation suggests that other CC surfaces are also important with perhaps having a role in interacting with the other flanking subdomains of sGC. Our results also extend beyond guanylyl cyclases as the CC structure is, to our knowledge, the first S-helix structure and serves as a model for all S-helix containing family members.

## Methods

### Cloning and mutagenesis of rsGCβ1 CC domain

The coding sequence (residues 348-409) for the CC region from rsGCβ1 was subcloned into the pET15b vector between Nde I and BamH I restriction sites using the following primer set: Forward primer: 5'-gga att cca tat ggc tac acg aga cct ggt cct ttt-3', backward primer: 5'-cgc gga tcc tca ctt gtg tct cag ctc att ggc aac-3'. The I371 M mutant was generated by site directed mutagenesis method, to facilitate structure determination by SeMet phasing (forward primer: 5'-ca caa gag ctg gaa atG ctc aca gac agg ctg c-3' and backward primer: 5'-g cag cct gtc tgt gag Cat ttc cag ctc ttg tg-3'). The resulting pET15b_rsGCβ1_348-409I371 M plasmid encodes the following polypeptides in the T7 expression region: mgsshhhhhhssglvpr/gshmATRDLVLLGEQFREEYKLTQELE**M**LTDRLQLTLRALEDEKKKTDTLLYSVLPPSVANELRHK (The residues that belong to rsGCβ1 are shown as uppercase letters. The single mutation was indicated in bold font. Other residues in lower case are introduced as cloning artifact.)

### Expression and purification of rsGCβ1 CC domain

The pET15b_rsGCβ1_348-409I371 M vectors were transformed into *E. coli *BL21 (DE3) pLysS cells (Invitrogen). The bacteria were grown in 8L M9 minimal media containing 50 μg/mL ampicillin and 37 μg/mL chloramphenicol at 37°C until a cell density of 1.2-1.4 OD_600 _was reached. The protein expression was induced with 300 μM isopropyl-β-D-thiogalactopyranoside (IPTG) supplemented with essential amino acids and selenomethionine for 8 hours at 37°C. The cells were harvested by centrifugation at 6000 rpm, followed by freezing at -80°C. The pellet was thawed on ice, resuspended in buffer A containing 20 mM Tris-HCl, 100 mM NaCl, 2 mM β-mercaptoethanol and lysed by sonication. The crude lysate was clarified by centrifugation at 16,000 rpm for 15 minutes, incubated with 3 mL Ni-NTA (Qiagen) for 4 hours and washed with buffer A plus 15 mM imidazole extensively. The protein was eluted using 50 mM buffer A plus 350 mM imidazole and dialyzed against 4L buffer A overnight. Thrombin (Sigma) digestion was carried out afterwards at 20°C for 8 hours and monitored by SDS-PAGE. The untagged protein product was loaded onto a Hitrap Q-sepharose (Amersham-Pharmacia Biotech) column and eluted with NaCl gradient. Fractions containing protein of interest were pooled together, concentrated and frozen in aliquots at -80°C. Prior to crystallization trials, a gel filtration polishing step using Superdex75 (Amersham-Pharmacia Biotech) was applied and the final protein buffer is 10 mM Tris-HCl, 100 mM NaCl, 2 mM β-mercaptoethanol.

### rsGCβ1 CC domain crystallization

X-ray diffraction-quality crystals of the CC construct were obtained at 20°C with the sitting-drop method by mixing 2 μl of the protein solution with 1 μl of the precipitant solution. Drops were equilibrated with a well containing 300 μl of 0.1 M Bis-Tris pH 7.0, and 0.7 M Ammonium Sulfate. Crystals of average dimensions of 300*20*20 μm^3 ^were grown in the above condition. For data collection at, the crystals were stepwise transferred into mother liquor reservoir solution with 5%, 10%, and 15% glycerol prior to freezing the crystal for data collection.

### rsGCβ1 CC domain structure determination and refinement

A Single Anomalous Dispersion (SAD) dataset for the rsGCβ1_348-409-I371 M crystal was collected at NSLS X29 beamline at Se peak wavelengths to 2.15 Å resolution and processed with HKL2000 [47]. The crystal belongs to space group C2, with cell dimensions a = 152.039 Å, b = 65.814 Å, c = 98.626 Å, β = 129.948° and eight molecules in the asymmetric unit. The program SOLVE/RESOLVE [48] was used to locate the selenium sites, calculate the experimental phases, and build an initial partial model. Refinement was carried out using REFMAC [49] alternated with rounds of manual model rebuilding and water picking using COOT [34]. The stereochemistry was checked using PROCHECK [50](Table 1). Figures are generated using Pymol http://pymol.sourceforge.net/. Coordinates and structure factors for the sGCβ1 CC domain have been deposited with the PDB (PDB identifier 3HLS).

## Abbreviations

sGC: soluble guanylyl cyclase; H-NOX: heme-nitric-oxide-and-oxygen binding domain (or H-NOB); H-NOXA: heme-nitric-oxide-and-oxygen binding associate domain (or H-NOBA); H-NOB: heme nitric oxide binding domain; H-NOBA: heme nitric oxide binding domain associated domain; CC: coiled-coil; GC: guanylyl cyclase; ECD: extra-cellular domain; TM: transmembrane helix; KHD: kinase-homology domain.

## Authors' contributions

XM designed and carried out the experiments and wrote the initial draft of the manuscript. AB helped plan the experiments and aided in the manuscript revisions. FVDA designed experiments, analyzed the data and helped write the manuscript. All authors read and approved the final manuscript.
